# Preliminary clinical observation of double C-arm digital subtraction angiography guidance during transjugular intrahepatic portosystemic shunt placement

**DOI:** 10.1186/s12876-023-02745-z

**Published:** 2023-04-06

**Authors:** Jianqiao Chen, Xiao Bai, Chunyan Wang, Jihua Li, Weiguo Xu

**Affiliations:** Zhuhai Hospital Affiliated With Jinan University (Zhuhai People’s Hospital), No. 79 Kangning Road, Zhuhai, 519000 Guangdong Province China

**Keywords:** Double C-arm DSA, Portosystemic shunt, Transjugular intrahepatic shunt, Portal hypertension

## Abstract

**Background:**

This study aimed to investigate the safety, preliminary clinical experience, and technical advantages of double C-arm digital subtraction angiography -assisted portal vein puncture for transjugular intrahepatic portosystemic shunt.

**Methods:**

Clinical data of 25 patients with portal hypertension caused by liver cirrhosis were retrospectively analyzed from January 2021 to June 2022. The fluoroscopy time, puncture time, mean portosystemic pressure gradient, dose area product, and intraoperative and postoperative complications were recorded.

**Results:**

Transjugular intrahepatic portosystemic shunt was performed in all 25 patients, with a success rate of 100%. The fluoroscopy time, puncture time, and dose area product were 33.6 ± 8.5 min, 9.1 ± 5.7 min, and 126 ± 53 Gy·cm^2^, respectively. The mean portosystemic pressure gradient decreased from 22.5 ± 6.3 mmHg to 10.5 ± 2.3 mmHg (*p* < 0.01). No serious intraoperative and postoperative complications were found.

**Conclusion:**

Double C-arm digital subtraction angiography-assisted portal vein puncture is safe and feasible in transjugular intrahepatic portosystemic shunt operation. It can reduce the difficulty of the operation and possesses evident technical advantages.

## Introduction

Transjugular intrahepatic portosystemic shunt (TIPS) has been widely used as a rescue treatment for esophagogastric variceal bleeding and as an early preventive treatment for secondary bleeding in complications related to portal hypertension. TIPS is the only effective relief method for patients with refractory ascites and pleural effusion [1–3]. Despite its recognized therapeutic efficacy, TIPS remains one of the most technically challenging procedures. The most time-consuming and challenging step is the establishment of hepatic and portal venous access, which is often the cause of TIPS failure [4].

We used double C-arm digital subtraction angiography (DSA) for TIPS treatment. This fluoroscopy unit consists of two C-arms that can be rotated and adjusted, allowing simultaneous observation in two directions. The observation plane is no longer a single two-dimensional perspective plane, and the position, direction, and depth of the puncture target are compared from two directions to adjust the needle path in real time and reduce the number of punctures. This new scheme reduces the difficulty of operation and improves the technical success rate and safety of TIPS. This study aimed to evaluate the clinical value of double C-arm DSA-assisted portal venous puncture in patients with portal hypertension and to provide new ideas and methods for clinical treatment.

## Methods

### Patient characteristics and design

This study was approved by the ethics committee of our hospital (Approval Number: 2020–75). The clinical data of 25 patients with liver cirrhosis and portal hypertension treated with TIPS from January 2021 to June 2022 were retrospectively collected from the electronic medical records of our hospital. All patients underwent double C-arm DSA-assisted TIPS in which the portal vein was punctured through the hepatic vein. All patients had a preoperative diagnosis of cirrhosis confirmed by laboratory tests and imaging such as ultrasonography, computed tomography (CT), and Magnetic Resonance imaging (MRI). Relative contraindications of liver puncture, particularly dysfunction of blood coagulation, were corrected with drugs or blood products before the operation. Patients with massive ascites underwent preoperative drainage via laparotomy. Although variceal bleeding is an indication, bleeding from abusing alcoholic beverages is a contraindication for this procedure. The baseline data and etiology of the patients are detailed in Table 1.

**Table 1:** Baseline data and etiology of 25 patients treated with TIPS

Characteristics of the patients	
Average age (years)	53.48 ± 8.15
Sex (no. of patients)	
Male	23 (92%)
Female	2 (8%)
Child–Pugh grade (no. of patients)	
Class A	9 (36%)
Class B	16 (64%)
Cause of cirrhosis (no. of patients)	
HBV	15 (60%)
HCV	2 (8%)
Alcoholic cirrhosis	7 (28%)
Unknown	1 (4%)
TIPS indications (no. of patients)	
Variceal bleed	15 (60%)
Refractory ascites	9 (36%)
Refractory pleural fluid	1 (4%)
Adjuvant embolization (no. of patients)	15 (60%)

*TIPS* Transjugular intrahepatic portosystemic shunt, *HBV* Hepatitis B virus, *HCV* Hepatitis C virus

### Equipment and materials

**Fig. 1:**
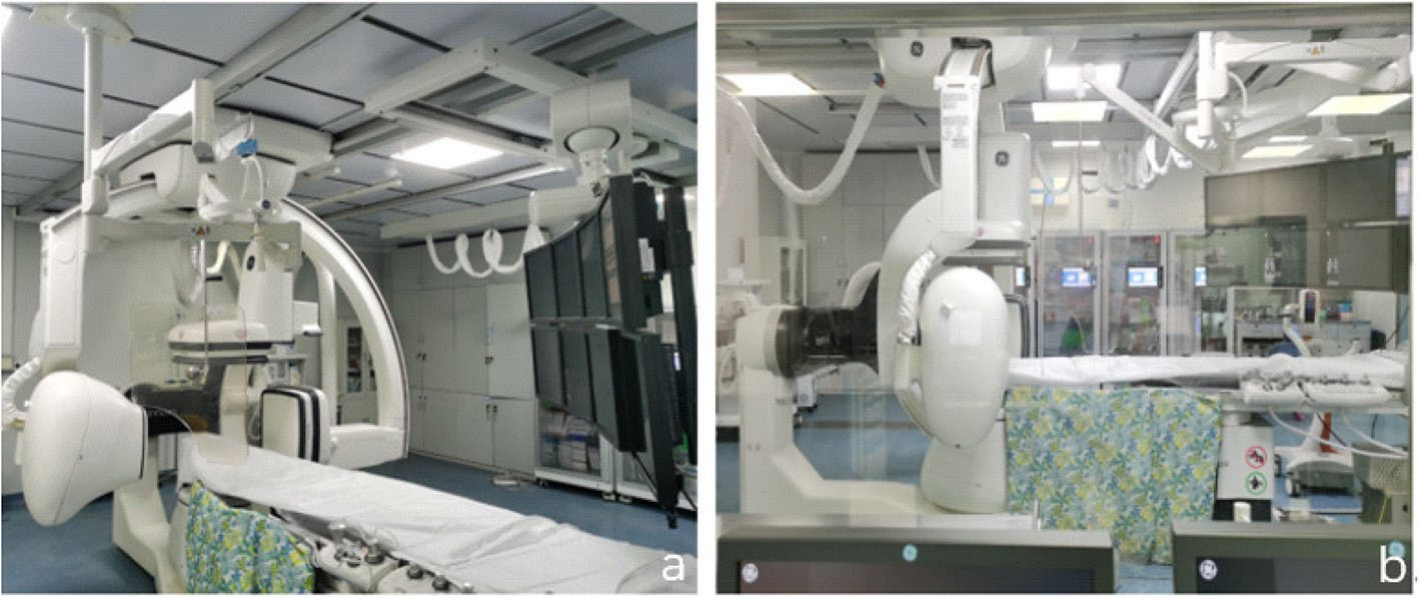
**a**, **b** The double C-arm digital subtraction angiography (DSA) system

Double C-arm DSA operating table (Innova IGS630; GE Healthcare, Chicago, IL, USA) (Fig. 1), Doppler ultrasound machine (Aplio 500; Toshiba Corporation, Tokyo, Japan), TIPS puncture device (Cook Medical, Bloomington, IN, USA), hardened guidewire (Amplatz; Boston Scientific, Marlborough, MA, USA), and Viatorr stent (Gore Medical, Flagstaff, AZ, USA) were used.

### TIPS procedure

**Fig. 2:**
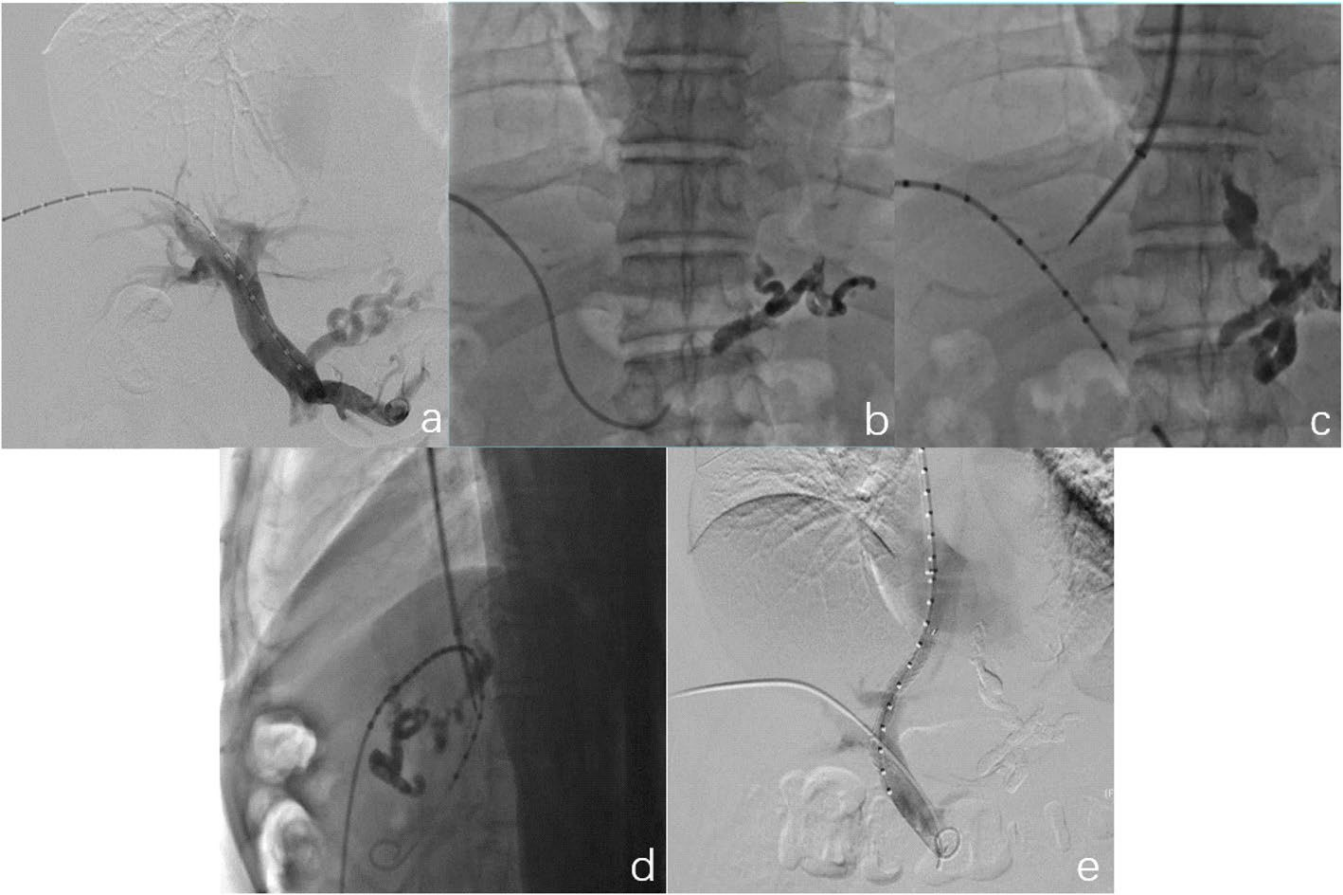
Portal venogram after successful percutaneous hepatic puncture portal vein placement (**a**). Intraoperative embolization of the thick left gastric vein was simultaneously performed (**b**). Portal vein puncture (towards the fourth puncture point) was guided by simultaneous observation in the frontal and lateral positions (**c**, **d**). After stent placement, re-imaging showed patency of the shunt (**e**)

Patients were placed supine on the double C-arm DSA operating table. They underwent surgery under general anesthesia, with routine disinfection of the right jugular vein and hepatic field skin and towel laying. Under ultrasound guidance, the portal vein branch (usually the right branch of the portal vein) was percutaneously punctured with a 21G Chiba needle. After successful puncture was confirmed by blood sampling, a marked pigtail catheter was distally inserted into the splenic vein. Portography was directly performed through a pigtail catheter (Fig. 2a). The puncture path was rationally planned by simultaneous observation in positive and lateral positions, and the puncture target was determined according to the marked points of the pigtail catheter.

Variceal embolization was performed if angiogram showed thickening or distortion of the esophagogastric venous plexus or if the patient had acute or short-term (within 2 weeks) bleeding prior to TIPS. The microcatheter was inserted to the target vein, which was embolized with a coil or a mixture of medical glue and lipiodol under fluoroscopy, and embolization was considered successful if no thickening or distortion of the plexus was observed on repeat imaging (Fig. 2b).

Under ultrasound guidance, the right internal jugular vein was punctured and a 5-F sheath was retained. Subsequently, a "mudskipper" guidewire was inserted into the inferior vena cava, and a 10-F sheath was exchanged to dilate the internal jugular vein puncture channel. A TIPS puncture set was then introduced through the guidewire to the intended hepatic vein branch (middle hepatic vein or right hepatic vein). The puncture was performed simultaneously and in real time in frontal and lateral positions under double C-arm DSA fluoroscopy against the most appropriate portal vein puncture target. The puncture path and angle could be adjusted in real time during the puncture process (Fig. 2c, d; the fourth marker point of the puncture site was determined for this patient). Once the entry of the puncture into the portal branch was successful by blood retrieval and contrast injection, measurements of portal venous pressure and body venous pressure were performed to determine the initial portosystemic pressure gradient. Subsequently, a stiffened guidewire was introduced, and the distal end was left in the superior mesenteric or splenic vein. A balloon (6–10 mm in diameter) was introduced through a guidewire and placed between the puncture channels of the portal and hepatic veins. The shunt tract was dilated, and the expansion process of the balloon was carefully observed. The balloon was then withdrawn, and a marked pigtail catheter was introduced. Imaging was performed to assess the length of the shunt channel (the above marked points allow accurate calculation of the shunt tract, each marked point being 1 cm apart). The Viatorr stent (8–10 mm in diameter) of appropriate length was selected and released, and the portal vein and body vein pressure were measured again. Re-imaging showed patency of the shunt (Fig. 2e). At the end of the TIPS procedure, the portal vein sheath was removed, and the puncture tract was sealed using gelatin sponge strips. All operations were performed by the same group of interventionalists.

### Postoperative treatment

Vital signs of the patients were monitored for 24 h after operation. Strict dietary control was implemented, and oral lactulose and oral ornithine aspartic acid were administered to prevent constipation and hepatic encephalopathy, respectively. Patients were discharged from the hospital for regular outpatient review, and if symptoms recurred, admission to the hospital was recommended for treatment and TIPS shunt recanalization, if necessary.

### Observation indicators and criteria

The technical success rate of dual C-arm DSA-assisted portal vein puncture, fluoroscopy time, portal vein puncture time, change in preoperative and postoperative portosystemic pressure gradient, dose area product (DAP), puncture complications associated with portal vein puncture, and complications and mortality within 6 months (mean median follow-up time) after the procedure were determined. Introducing the guidewire through the hepatic vein into the main portal vein was labeled a technical success. Procedural complications were recorded, including hepatic artery injury, biliary tract injury, and abdominal bleeding. Postoperative complications include hepatic encephalopathy, shunt channel blockage, and liver failure.

### Statistical analyses

Statistical analysis was performed with SPSS 25.0 software (version 25.0; IBM, Armonk, NY, USA). Data are expressed as mean ± standard deviation by using the t-test or ANOVA. Statistical significance was set at *p* value < 0.05. Count data are expressed as composition ratios or percentages.

## Results

### Interventional results

All 25 TIPS patients were treated with a portal shunt tract, with a 100% technical success rate. A total of 25 stents were intraoperatively inserted. The fluoroscopy time, puncture time, and DAP were 33.6 ± 8.5 min, 9.1 ± 5.7 min, and 126 ± 53 Gy·cm^2^, respectively. The portosystemic pressure gradient decreased from 22.5 ± 6.3 mmHg to 10.5 ± 2.3 mmHg before surgery (*p* < 0.01). The difference in portal vein pressure was significantly reduced and statistically significant. No intraoperative puncture-related complications, such as hepatic artery injury, biliary tract injury, and abdominal bleeding, were observed in any patient. Postoperative hepatic puncture tract embolization was routinely performed with sponge strips, and no abdominal bleeding was observed in any patient.

### Recent follow-up results

Fifteen (60%) patients with esophagogastric fundic varices had combined gastroesophageal variceal vein embolization with TIPS, and no patients developed or redeveloped ruptured variceal bleeding during the postoperative follow-up period. Patients with refractory ascites and hydrothorax were treated with postoperative drainage diuresis, which significantly reduced their symptoms. One patient developed hepatic encephalopathy 3 days postoperatively, and the patient improved after 3 days of acetic acid enema and ornithine aspartic acid injection 7.5 g qd. The remaining patients did not develop postoperative complications, such as hepatic encephalopathy and shunt channel blockage, during the median follow-up period of 6 months and recovered well after surgery. These results are summarized in Table 2.

**Table 2:** Interventional and follow-up result

Parameters		*P*—value
Technical success rate	100%	
Fluoroscopy time (min)	33.6 ± 8.5	
Puncture time (min)	9.1 ± 5.7	
DAP (Gy·cm2)	126 ± 53	
Pressure gradient (mmHg)		0.01
Pre-TIPS	22.5 ± 6.3	
Post-TIPS	10.5 ± 2.3	
Stent diameter (mm)	8	
No. of stents placed	25	
Intraprocedural complication (no. of patients)		
Hepatic artery injury	0	
Biliary tract injury	0	
Abdominal bleeding	0	
Postoperative complications (no. of patients)		
Hepatic encephalopathy	1	
Shunt channel blockage	0	
Hospital stay duration (days)	5.5 ± 0.7	

## Discussion

More than 90% of patients with cirrhosis and portal hypertension develop intractable ascites or recurrent variceal rupture bleeding [2]. TIPS offers a less invasive and faster recovery option for patients with complications due to portal hypertension [5].

Traditional TIPS treatment mostly uses the superior mesenteric or splenic artery angiography for indirect visualization of the portal vein. It requires patients to undergo breath holding training, which makes it difficult for elderly patients or those with poor lung function to cooperate. This results in unsatisfactory image quality and therefore a large degree of blindness, leading to repeated multiple punctures, prolonged procedure, and increased risk of radiation exposure. Complications of TIPS are directly related to the number of punctures, and an increase in the number of punctures leads to a higher incidence of complications. Hepatic and biliary artery injuries can occur in up to 6% and 5% of cases, respectively [6]. Therefore, accurate location of the target site of portal vein puncture is particularly important to improve the accuracy of the operation and reduce the number of punctures. In the more than 40 years of TIPS development, new adjunctive puncture methods have emerged clinically, and the puncture procedure has become more precise and safer. These methods include CO_2_ wedge portal venography, transabdominal ultrasound [7], intravascular ultrasound [8], portal vein placement marker targets [9, 10], and 3D image fusion navigation with CT or MRI [11, 12].

Wedge portal venography is the most commonly used method to identify the portal vein. CO_2_ as a contrast agent is significantly better than iodine for portal imaging. It reduces potential nephrotoxicity and the incidence of postoperative nephropathy. However, a risk of infarction and hepatic parenchymal laceration during pushing remains [13]; prolonged arterial CO_2_ stasis may produce tissue ischemia, and static CO_2_ may also produce vasoconstriction [14]. Relevant studies showed that transabdominal ultrasound and intravascular ultrasound-assisted portal vein puncture are significantly superior to the conventional TIPS approach in terms of radiation exposure [7, 15]. The puncture process can be monitored in real time with real-time adjustment of the needle direction and angle, which can theoretically point to the optimal target for portal vein puncture. However, both methods are limited by proficiency in professional skills and require the presence of an operator familiar with ultrasound anatomical plane during the puncture. Moreover, image quality tends to be poorer in obese patients and in those with more intestinal gas. Additional equipment increases the consumables for the procedure and the cost of the procedure to the patient, and the learning curve for the operator is higher, leading to a higher level of complexity.

In recent years, multimodal medical image fusion has become a relatively new field of research. It is increasingly used in interventional procedures, where 3D images are fused into DSA 2D real-time fluoroscopic images to navigate portal vein punctures with much higher efficiency. In the study of Meine et al., the advantages of fusion technology for less-experienced interventionalists were particularly emphasized [16]. Our participants (*n* = 5) also included three interventionalists with less TIPS experience. We received positive feedback from them, reducing the difficulty of establishing access. Although technology and algorithm changes are constantly being used to increase the accuracy of image matching [16–18], this can still lead to poor image matching and partial discrepancies in the fused images due to patient position, breathing, and mechanical pressure during needle insertion, causing liver displacement. In a study of multimodal image fusion DSA for TIPS procedure, 44% of the patients (8/18) showed image mismatch, mainly due to the patient's arm position, large amounts of ascites, and respiratory motion [17]. This fusion requires complex post-3D reconstruction techniques and depends heavily on technician post-processing.

By contrast, all 25 patients in this study underwent assisted portal vein puncture using double C-arm DSA. The portal vein was initially punctured using ultrasound-guided percutaneous transhepatic puncture. Direct imaging of the portal vein was performed using double C-arm DSA fluoroscopy for dual-angle visualization. Double C-arm DSA has been used in neurointerventions and orthopedic interventions [19, 20]. It improves the precision of the procedure and reduces the operation and radiation times.

The use of double C-arm DSA in TIPS surgery has several advantages. First, ultrasound-guided transabdominal puncture of the portal vein and retention of a pigtail catheter with marker points allow direct imaging of the portal vein, obtaining clear images of the portal vein and reducing complication rates due to blind puncture. Second, after a successful ultrasound-guided puncture of the portal vein, varicose vein embolization can be performed through this access for emergency hemostasis or prevention of rebleeding, which is more convenient and reduces the time to perform superselection. With TIPS alone, although the shunt obstruction is relieved, the varicose plexus remains and the risk of bleeding is 20%-30% [21, 22]. In a retrospective study investigating adjuvant embolization therapy, the rebleeding rate was close to 6% at a median follow-up of 26 months and 1% and 3% at 1 and 2 years, respectively [23]. In our study, 60% of the patients (15/25) underwent combined variceal embolization during TIPS, and the patients had no postoperative discomfort and no recurrent rupture bleeding during the follow-up period (mean, 6 months). Third, intraoperative fluoroscopy is often performed in different directions. Using a dual C-arm to perform fluoroscopy in two directions simultaneously eliminates the need to repeatedly adjust a single fluoroscopic C-arm. This saves operation time, thereby reducing the risk of postoperative infection and shortening the patient's recovery time. Fourth, simultaneous monitoring in the frontal and lateral positions of both C-arms allows for better evaluation of patients with complex portal vein anatomy. In cases where the operation is difficult, such as severe cirrhosis resulting in an atrophied liver, or patients with bare leakage of the main trunk and secondary branches of the portal vein outside the liver, individualized puncture protocols can be developed, which greatly broadens the indications for TIPS. Fifth, better clarification of the target point of puncture, puncture towards the target point of the pigtail catheter according to the real-time ortholateral images, better control of the angle, depth, and direction of needle entry, and real-time adjustment when the needle path deviates from the target point result in the achievement of a fixed-point puncture, decreased mechanical damage to the liver, and no intraoperative puncture-related complications in all patients. Sixth, the operation is simple, and the learning curve is low, which lowers the threshold for performing TIPS operation and is more suitable for beginners. Finally, the recent efficacy is satisfactory, and the prognosis of all patients is related to their liver reserve capacity. All patients in this group had grade A or B liver function, and the symptoms of portal hypertension were effectively controlled postoperatively.

Given the retrospective nature of the study, the recording of the number of puncture needles may be subject to recall bias. We assessed the number of puncture needles by fluoroscopy time and puncture time, which were used as indirect indicators of the number of puncture needles. The current study was conducted in a single institution, and the sample size was relatively small, lacking comparative data. A large sample size of randomized controls is still warranted for follow-up. In conclusion, this method of assisted portal venipuncture TIPS, which provides clear guidance during puncture, improves accuracy and is simpler, safer, and more effective than conventional TIPS procedures. Moreover, it has reduced intraoperative complication rate, has obvious advantages, and deserves clinical promotion.

## Data Availability

The data used to support the findings of this study are available from the corresponding author on reasonable request.

## References

[1] Horhat A, Bureau C, Thabut D, Rudler M (2021). Transjugular intrahepatic portosystemic shunt in patients with cirrhosis: Indications and posttransjugular intrahepatic portosystemic shunt complications in 2020. United Eur Gastroenterol J.

[2] Allaire M, Walter A, Sutter O, Nahon P, Ganne-Carrié N, Amathieu R (2020). TIPS for management of portal-hypertension-related complications in patients with cirrhosis. Clin Res Hepatol Gastroenterol.

[3] Strunk H, Marinova M (2018). Transjugular intrahepatic portosystemic shunt (TIPS): Pathophysiologic basics, actual indications and results with review of the literature. RoFo.

[4] Isfort P, Penzkofer T, Wilkmann C, Na HS, Kotzlowski C, Ito N (2017). Feasibility of electromagnetically guided transjugular intrahepatic portosystemic shunt procedure. Minim Invasive Ther Allied Technol.

[5] Rajesh S, Philips CA, Betgeri SS, George T, Ahamed R, Mohanan M (2021). Transjugular intrahepatic portosystemic shunt (TIPS) placement at index portal hypertensive decompensation (anticipant TIPS) in cirrhosis and the role of early intervention in variceal bleeding and ascites. Indian J Gastroenterol.

[6] Gaba RC, Khiatani VL, Knuttinen MG, Omene BO, Carrillo TC, Bui JT (2011). Comprehensive review of TIPS technical complications and how to avoid them. AJR Am J Roentgenol.

[7] Cam I, Gencturk M, Shrestha P, Golzarian J, Flanagan S, Lim N (2021). Ultrasound-guided portal vein access and percutaneous wire placement in the portal vein are associated with shorter procedure times and lower radiation doses during TIPS placement. AJR Am J Roentgenol.

[8] Pillai AK, Andring B, Faulconer N, Reis SP, Xi Y, Iyamu I (2016). Utility of intravascular US-guided portal vein access during transjugular intrahepatic portosystemic shunt creation: Retrospective comparison with conventional technique in 109 patients. J Vasc Interv Radiol.

[9] Chen Y, Ye P, Li Y, Ma S, Zhao J, Zeng Q (2015). Percutaneous transhepatic balloon-assisted transjugular intrahepatic portosystemic shunt for chronic, totally occluded, portal vein thrombosis with symptomatic portal hypertension: Procedure technique, safety, and clinical applications. Eur Radiol.

[10] Teitelbaum GP, Van Allan RJ, Reed RA, Hanks S, Katz MD (1993). Portal venous branch targeting with a platinum-tipped wire to facilitate transjugular intrahepatic portosystemic shunt (TIPS) procedures. Cardiovasc Intervent Radiol.

[11] Chivot C, Robert B, Bouzerar R, Popoff R, Yzet T (2018). 3D C-Arm cone beam CT for targeting the portal vein during TIPS: Initial clinical experience. Eur J Radiol.

[12] Kee ST, Ganguly A, Daniel BL, Wen Z, Butts K, Shimikawa A (2005). MR-guided transjugular intrahepatic portosystemic shunt creation with use of a hybrid radiography/MR system. J Vasc Interv Radiol.

[13] Semba CP, Saperstein L, Nyman U, Dake MD (1996). Hepatic laceration from wedged venography performed before transjugular intrahepatic portosystemic shunt placement. J Vasc Interv Radiol.

[14] Johnson PL, Neperud J, Arnold J, Thomas J (2011). Livedo reticularis and bowel ischemia after carbon dioxide arteriography in a patient with CREST syndrome. J Vasc Interv Radiol.

[15] Kao SD, Morshedi MM, Narsinh KH, Kinney TB, Minocha J, Picel AC (2016). Intravascular ultrasound in the creation of transhepatic portosystemic shunts reduces needle passes, radiation dose, and procedure time: A retrospective study of a single-institution experience. J Vasc Interv Radiol.

[16] Meine TC, Dewald CLA, Becker LS, Mähringer-Kunz A, Massoumy B, Maschke SK (2020). Transjugular intrahepatic portosystemic shunt placement: Portal vein puncture guided by 3D/2D image registration of contrast-enhanced multi-detector computed tomography and fluoroscopy. Abdom Radiol (NY).

[17] Rouabah K, Varoquaux A, Caporossi JM, Louis G, Jacquier A, Bartoli JM (2016). Image fusion-guided portal vein puncture during transjugular intrahepatic portosystemic shunt placement. Diagn Interv Imaging.

[18] Luo X, Wang X, Yu J, Zhu Y, Xi X, Ma H (2018). Transjugular intrahepatic portosystemic shunt creation: three-dimensional road map versus CO2 wedged hepatic venography. Eur Radiol.

[19] Çelik H, Kara A, Sağlam Y, Türkmen İ, Aykut S, Erdil M (2018). Can double fluoroscopy in the oblique position reduce surgical time and radiation exposure during intertrochanteric femur fracture nailing?. Ulus Travma Acil Cerrahi Derg.

[20] Kara A, Celik H, Seker A, Uzun M, Sonmez MM, Erdil M (2016). Procedural outcomes of double vs. single fluoroscopy for fixing intertrochanteric femur fractures. Arch Orthop Trauma Surg..

[21] Lv Y, Yang Z, Liu L, Li K, He C, Wang Z (2019). Early TIPS with covered stents versus standard treatment for acute variceal bleeding in patients with advanced cirrhosis: a randomised controlled trial. Lancet Gastroenterol Hepatol.

[22] Lv Y, Qi X, He C, Wang Z, Yin Z, Niu J (2018). Covered TIPS versus endoscopic band ligation plus propranolol for the prevention of variceal rebleeding in cirrhotic patients with portal vein thrombosis: a randomised controlled trial. Gut.

[23] Schultheiß M, Giesler M, Maruschke L, Schmidt A, Sturm L, Thimme R (2019). Adjuvant transjugular variceal occlusion at creation of a transjugular intrahepatic portosystemic shunt (TIPS): Efficacy and risks of bucrylate embolization. Cardiovasc Intervent Radiol.

